# Preparing Midwifery Students for the Certification Examination by Debunking Prevalent Test‐Taking Myths

**DOI:** 10.1111/jmwh.70016

**Published:** 2025-08-26

**Authors:** Kendra Faucett

**Affiliations:** ^1^ Vanderbilt School of Nursing Nashville Tennessee

**Keywords:** cognitive psychology, certification exam, heuristics, metacognition, midwifery education, quality improvement, test‐taking tips

## Abstract

Nationally, midwifery educators are often perplexed when graduates from their programs are not successful on the American Midwifery Certification Board (AMCB) certification examination. Often, these students are excellent clinically and have a deep foundational knowledge. During examination reviews with professors, they may be able to explain a concept well orally but struggle to pick the correct answer. Emerging cognitive science can be applied to test‐taking heuristics to better understand how students make choices on multiple‐choice tests. Helping students understand and develop metacognition and using a trauma‐informed pedagogy will give educators a new lens for teaching test‐taking strategies. By applying a new method, midwifery educators can dispel common test‐taking myths, help their students understand their cognitive bias when taking tests, and ultimately build students’ confidence when approaching high‐stakes tests. By changing their approach to test‐taking advice, educators can help more students pass the certification on the first attempt and increase the speed at which new graduates are ready to enter the workforce.

## INTRODUCTION

To become a Certified Nurse‐Midwife (CNM) or Certified Midwife (CM) in the United States, a candidate must obtain either a Master's Degree, a Post‐Master's Certificate, or a Doctorate of Nursing Practice from a midwifery program at an education institution accredited by the Accreditation Commission for Midwifery Education.^1^, ^2^, ^3^ The eligible graduate must pass the American Midwifery Certification Board (AMCB) certification examination to earn the CNM or CM credential.^2^ Each CNM or CM then seeks licensure in their chosen state of practice. The board examination or “the boards,” as the AMCB certification examination is commonly known, is the gateway to practicing midwifery in the United States. Graduates apply to the AMCB for eligibility to take the examination, pay a $500.00 application fee, and schedule their examination through a national testing service.^2^ The test has 175 multiple‐choice questions with 4 possible answer choices.^2^

Although the AMCB is the only certifying body for midwifery, other disciplines have multiple certifying bodies. For midwives in 2024, the national AMCB first‐time pass rate was 80%, down 4% from 2023.^4^, ^5^ Midwifery first‐time board pass rates are among the lowest of various specialties (see Table 1).

**Table 1:** A Comparison of National First‐Time Certification Examination Pass Rates by Advanced Practice Specialties

Advanced Practice Specialty	Certifying Body	2024 National First‐Time Pass Rate, %	2023 National First‐Time Pass Rate, %	2022 National First‐Time Pass Rate, %
Midwifery	AMCB	80^4^	84^5^	83^43^
Women's Health Gender‐related	NCC	79^44^	87^44^	89^44^
Family Nurse Practitioner	AANP	83^45^	73^46^	74^47^
	ANCC	83^48^	85^49^	86^50^
Psychiatric Mental Health	AANP	82^45^	n/a	n/a
	ANCC	83^48^	90^49^	n/a
Emergency	AANP	91^45^	86^46^	89^47^
Neonatal	ANCC	89^48^	81^49^	83^50^

Abbreviations: AANP, American Academy of Nurse Practitioners certification board; AMBC, American Midwifery Certification Board; ANNC, American Nurses Credentialing Center; n/a, not applicable; NCC, National Certification Corporation.

With the decline in the national AMCB first‐time pass rate, many midwifery educators are eager for strategies to help their students be successful on their first attempt.^4^ A candidate is allowed a maximum of 4 attempts.^2^ If they are unsuccessful after 4 attempts or within 2 years of graduation, they must repeat a midwifery program.^2^ Candidates who do not pass the examination on the first attempt have a statistically lower chance of passing the remaining 3 attempts.^5^ Many midwifery programs offer their students a formative or summative 175‐question mock board examination to help students gauge readiness to take the certification examination. It is important that educators ensure that the examination is up‐to‐date with current recommendations, follows the AMCB examination blueprint and task analysis, and is valid and reliable.^2^, ^6^

From formal, comprehensive review courses within a midwifery graduate program to focused workshops through the American College of Nurse‐Midwives, the author has prepared over a thousand students for the board examination. Insights from hundreds of one‐on‐one test reviews with students and a deep interest in cognitive psychology and heuristics suggest that several common test‐taking tips do not necessarily apply to a diverse midwifery student population. Four common test‐taking myths are explored, including relevant data from cognitive neuroscience, rationales to debunk each myth, and new recommendations for midwifery faculty.

Continuing education (CE) is available for this article. To obtain CE online, please visit http://www.jmwhce.org. A CE form that includes the test questions is available in the print edition of this issue.

## COMMON TEST‐TAKING TIP MYTHS

Students are inundated with test‐taking tips when searching Google or social media. When searching Google with phrases like “nursing test‐taking tips,” “graduate nurse test‐taking tips,” or “standardized exam test‐taking tips,” students will find many websites, blogs, discussion forums, and YouTube videos.^7^, ^8^, ^9^, ^10^, ^11^, ^12^, ^13^

**QUICK POINTS**

✦ The American Midwifery Certification Board certification examination is the final step for a graduate to earn the certified nurse‐midwife or certified midwife credential in the United States.
✦ The 2023 national board first‐time pass rate was 84%, declining to 80% in 2024.
✦ Some widely used test‐taking strategies stem from cognitive biases, which can impair decision‐making.
✦ Understanding neuropsychology, heuristics, and cognitive biases helps educators better serve their students.

### Myth 1: Take Boards as Soon as Possible

Students often rush to take the board examination as soon as they finish their program. They feel pressure to test while information is fresh in their minds and are eager to begin working as midwives.

#### Faculty Observations and Neurocognitive Science

Although students may feel pressure to test as soon as possible, major life events can be a heavy cognitive burden for even the most prepared student. Midwives, by nature, are tenacious and resilient. Interestingly, the same characteristics that successfully propel midwives through grueling hours, physical and mental demands, and emotional highs and lows of practice can be precisely why a new graduate is unsuccessful on the board examination. Many students have balanced working full‐time, raising children, or caring for parents, all while completing graduate school. They have grit in the face of adversity.^6^ During a significant life event, tenacious midwifery students think, “I will just go ahead and schedule boards and get it over with!”

In this scenario, resilient graduates use biased interpretation.^14^ ^(p101)^ This form of confirmation bias happens when they interpret new data (new major life events) to fit with what they think is the truth (“I always overcome obstacles”). This rationale may have applied to a 50‐question test during their education program, but it is likely impossible with a 175‐question, highest‐stakes certification examination.

#### New Recommendation

Faculty should advise graduates to take the boards when fully prepared and they have no acute life events occurring. Meeting with students individually to inquire about their postgraduation plans can provide faculty with relevant information to advise the student on board examination timing. Reviewing mock board examination scores and giving a reference point to a typical score needed to pass the examination can be helpful because this provides objective data. If a student needs to reschedule because of life circumstances or needs additional preparation, they may notify PSI examination services, the certification exam vendor, without penalty within 2 business days of their scheduled examination.^2^ Ideally, midwifery faculty could maintain points of contact postgraduation for continued support.

### Myth 2: Go With Your Gut

This is one of the most ubiquitous myths found on social media.^7^, ^8^, ^9^ Well‐meaning educators may also be asking students to “go with their gut” or, in other words, to trust themselves.^15^

#### Faculty Observations and Neurocognitive Science

Students with anxiety, depression, or a history of trauma may lack self‐trust and therefore, the ability to “go with their gut.” Self‐trust is a sign of mental health and a well‐adapted adult.^16^

Trauma is a result of events or circumstances that an individual feels were emotionally or physically harmful or a threat to their safety.^17^ The long‐lasting effects interfere with the person's mental, emotional, physical, or spiritual functioning and well‐being.^17^ Some midwifery students have experienced childhood, racial, academic, emotional, or other trauma that could affect their ability to learn.^18^, ^19^ As a result of trauma, students often internalize the message that they are “stupid” or “can't ever make a good choice.”^19^ Experiencing scarcity of “respect, esteem, safety, and acceptance” uses mental bandwidth and is exhausting.^20^

Trauma can potentially rewire the brain, specifically the amygdala, hippocampus, and prefrontal cortex.^21^ Neurotransmitters, such as cortisol and norepinephrine, are activated with the stress response and can affect memory.^21^ As early as 1995, Goleman coined the phrase “amygdala hijack” to describe the hypersensitive amygdala response.^22^ When confronted with a perceived threat, the overactive amygdala can trigger an alarm response, which initiates a physiologic stress response leading to fear.^22^, ^23^, ^24^ This response, by design, interrupts critical reasoning, working memory, and executive function within the prefrontal cortex.^24^ Brain rewiring after trauma can change how those exposed to trauma distinguish between what is safe and what is not.^25^ The signaling between the hippocampus (emotion and memory) and the salience network (for learning and survival) decreases.^26^ The perceived threat of the board examination can cause students with a history of anxiety or trauma to activate a complete stress response in the brain, leaving graduates disconnected from the prefrontal cortex.

If a student with a history of trauma is struggling with a question, simply telling them to “go with their gut” is not helpful. Victims of trauma often doubt their gut instincts.^16^, ^19^ If students have experienced a significant betrayal or shame in their past, they may default to thinking a test question is trying to trick them because they lack self‐trust.^16^ As their anxiety grows, it may present as abdominal pain or other somatic responses.^27^ Their self‐trust deficit can impair effective action.^16^

Likewise, people from historically marginalized or underrepresented groups (eg, multilingual, a person of color) commonly code‐switch or act differently to fit in with the dominant White culture.^28^ Code‐switching, identity shifting, or double consciousness is “altering one's actions, perspectives, appearance, and speech to mitigate negative outcomes of discrimination.” ^20^, ^29^ Their gut may tell them they are uncomfortable or unsafe, but they have had to ignore it as a coping strategy for career advancement or even for safety.^27^ A graduate with a history of trauma may perceive the high‐stakes certification examination as a threat to their survival and therefore their safety. This perceived threat can decrease a student's cognitive capacity and executive control, both of which are necessary for cognitive activities like an examination.^20^, ^23^, ^30^

#### New Recommendation

Educators need to use trauma‐informed educational practices.^18^, ^31^ Educators are encouraged to routinely examine their own implicit biases in their pedagogy and their institutions while working to learn microintervention strategies.^20^, ^32^, ^33^, ^34^ Faculty are encouraged to check program examination items for potential bias, including irrelevant difficulty, linguistic complexity, and cultural bias.^6^ Educators can also provide feedback to key stakeholders in their sphere of influence, leading to systemic changes in the test formats and administration. ^34^, ^35^ At the institutional level, midwifery faculty can advocate for having a teaching presence in foundational courses to influence those examination items. They can advocate for workload effort to reflect the time requirement for individual student remediation.

Faculty should recommend that students always use the blank sheet of paper given at the testing site. Students should write down memorized information before reading the first question. With the increased cortisol, a student may have difficulty accessing the prefrontal cortex.^24^ Having the information already noted can help overcome an amygdala hijack.^22^, ^24^, ^26^

### Myth 3: Don't Change Answers

Students routinely hear this advice from social media and educators alike.^10^, ^12^, ^13^, ^15^, ^36^

#### Faculty Observations and Neurocognitive Science

When students are unsure of an answer they selected, they are not only trying to think critically about the content in the stem of the question but also are debating whether to change their answer. Students commonly blame a low test score on “those 2 questions I changed.” For example, on a 40‐question test, a student must get 32 questions correct to earn an 80% passing grade. Often, students will miss 9 questions, just 1 more than permitted to pass the test, for 31 out of 40 (77.5%). When reviewing the test, students frequently lack the metacognition to realize they failed it because they missed 9 questions. Metacognition is generally defined as the ability to think about one's thinking and involves reflecting, understanding, and controlling one's learning.^14^, ^37^ Students become fixated on the 2 answers they may have changed from correct to incorrect and fail to recognize that they have not mastered the content of the 7 other questions, including the 2 they changed. They replace the correct thought, “I haven't fully mastered the content in this exam,” with the incorrect thought, “I should never change my answers.” Research on answer‐changing behaviors finds that students change answers from incorrect to correct the majority of the time.^15^, ^38^, ^39^, ^40^ Changing answers results in a net positive benefit in overall test scores.^15^, ^38^, ^39^, ^40^

When students decide whether to change an answer, this can lead to overthinking and rumination.^14^ Cognitive science shows this does not help with problem‐solving, nor does it help people discover solutions; instead, it leads to uncertainty and anxiety.^14^ Negativity bias indicates that people tend to remember negative things over positive things.^14^ Students tend to remember answers they changed from right to wrong because it is painful, and therefore, it has more weight in their memory.^14^, ^15^, ^39^ They simultaneously fail to recall instances in which they picked the incorrect answer confidently or changed an answer from incorrect to correct.^39^ The finding that students are reluctant to change answers against their first instinct is known as the “first instinct fallacy.”^39^

#### New Recommendation

Answer‐changing behaviors in students can be very nuanced and should be tailored to the individual student.^15^, ^36^, ^40^ Educators can help anxious or lower‐performing graduates eliminate this “change or not to change” agony. Students who struggle with confidence and rumination should flag the question relatively quickly and not mark an answer. They need to complete the entire examination and return to the flagged items. They can then review the question, spend time on their diagnostic reasoning, commit to an answer, and proceed to the next item. Faculty should review all questions with students during one‐on‐one examination reviews, not just missed items. Faculty are reinforcing the instinct fallacy by only focusing on the missed items.^39^

### Myth 4: Read the Question and Formulate an Answer Before Reading the Answer Options

This common tip is also phrased as “try answering the question before you read answer options” or “answer the question first.” ^8^, ^13^, ^42^. This myth suggests that a candidate should read the question and consider how they would answer before looking at the options. They then look at the answer options and hopefully find their formulated answer mentally.

#### Faculty Observations and Neurocognitive Science

There are 3 possible outcomes when using this strategy: (1) the answer the student thought of is a choice, they pick it, and it is correct; (2) the answer they thought of is a choice, they pick it, and it is incorrect, and (3) the answer they thought of is not a choice. Option 3 usually leads to anxiety, and the student must now reread the question with extra cortisol circulating. This tip is a waste of time and an easy way to default erroneously to confirmation bias.^14^ Confirmation bias is the tendency to stick with our preexisting beliefs.^14^ We look for evidence to confirm our suspicions when we think we know something.^14^

Reading a question first can lead someone to employ the framing effect, use the recency heuristic, or fall prey to “the curse of knowledge” problem.^14^ People unknowingly use the framing effect when they base their choices on how the options are framed rather than the options themselves.^14^ ^(p158)^ For example, a question might have several data points about blood pressure in the stem when it is actually about a routine screening colonoscopy. The question leads the test taker down the hypertension path, which may distract the student from noticing a critical detail (the patient's age) that warrants ordering a routine screening test.

The recency and the availability heuristics involve remembering an event we most recently experienced or the availability of information in our minds.^14^ ^(p.93,130)^ If students have recently studied a concept, experienced it in the clinical or work setting, or have committed it to memory, they often jump at opportunities to display that information. In doing so, they frequently fail to understand what the question was asking and instead hold a bias toward a particular answer because it was most available in their memory. This can even happen if a student has a previous question about a topic such as hormone therapy. In a subsequent question, the student is more likely to answer “hormone therapy” because the former question primed their brain to think about hormone therapy. Finally, the “curse of knowledge” concept stems from an egocentric bias.^14^ ^(p.198)^ Often, students remember and draw on topics from practice questions they recently completed. They may have worked in a specialized clinical setting or have personal experience with a specific obstetric or gynecologic topic. Once students have this specialized knowledge, they cannot imagine that other people don't have that knowledge. This leads them to overthink a question or add a backstory to the stem of a question.

#### New Recommendation

Educators should have students read the answer choices before reading the question item. The student can get an idea of what the question is asking based on the answer options provided. With that narrowed framework, the student reads the question through a more focused lens. This strategy makes it easier for the student to identify relevant and irrelevant information in the question stem.

To help students work through this strategy, faculty can help students analyze the answer choices. Students should have a strong idea of the question topic by simply looking at the answers provided. There are many ways to view the options, including but not limited to the level of midwifery intervention of each choice, appropriateness of a medication, or safety priority. One answer choice can often be eliminated before even reading the question because it is not a possibility in any scenario. Faculty can guide students to look for relevant clues in the question now that they know all possible answer choices. Once they have read the question, they may hypothesize that answer B is correct. Before selecting option B, the students must attempt to disconfirm their hypothesis. This requires thinking through the rationales for why the other 3 answer choices are incorrect. They can verbalize to the faculty member, “Option A is wrong because…” and in this manner provide rationales for or against each answer option. This is known as violating the hypothesis.^14^ Speedy test takers often skip this vital step, leading to a careless mistake.

## CONCLUSION

Midwives offer individualized care to clients and value the hallmark of shared‐decision‐making.^1^ The same is true for midwife educators and their students. Giving the best advice to new graduates involves knowing and understanding them as individuals and making honest, culturally sensitive, and trauma‐informed recommendations accordingly.

When educators meet with students to review tests, having the students talk through their heuristics or how they made those choices is fascinating and informs further advice. Instead of having the narrow focus of finding the questions they missed and explaining the rationale for the correct answer, educators have the opportunity to better guide students by questioning their thought processes on all examination items. The educator will likely learn as much, if not more, than the student. These recommendations, grounded in neuropsychology, can set students up for success as they approach their first attempt at the board examination, thereby allowing more midwives to enter clinical practice sooner.

## CONFLICT OF INTEREST

The author has no conflicts of interest to disclose.

## References

[1] Standards for the Practice of Midwifery . American College of Nurse‐Midwifery; 2022. Accessed Febuary 23, 2025. https://midwife.org/wp‐content/uploads/2024/10/Standards‐for‐the‐Practice‐of‐Midwifery.pdf

[2] AMCB Certification Exam Candidate Handbook Nurse‐Midwifery and Midwifery . American Midwifery Certification Board; 2025. Accessed May 10, 2025. https://www.amcbmidwife.org/amcb-certification/candidate-handbook

[3] Criteria for Programmatic Accreditation of Midwifery Education Programs With Instructions for Elaboration and Documentation . Accreditation Commission for Midwifery Education; 2020. Accessed Febuary 23, 2025. https://theacme.org/wp‐content/uploads/2023/08/Exhibit‐I‐Criteria‐for‐Programmatic‐Accreditation‐FinalCopyright‐May‐2019‐Revised‐August‐2020‐22.pdf

[4] American Midwifery Certification Board 2024 Annual Report . American Midwifery Certification Board; 2025. Accessed April 28, 2025. https://www.amcbmidwife.org/docs/default‐source/annual‐reports/2024‐amcb‐annual‐report.pdf

[5] American Midwifery Certification Board 2023 Annual Report . American Midwifery Certification Board; 2024. Accessed February 23, 2025. https://www.amcbmidwife.org/docs/default‐source/annual‐reports/2023‐amcb‐annual‐report.pdf

[6] Billings DM , Halstead JA , eds. Teaching in Nursing: A Guide for Faculty. 6th ed. Elsevier; 2020.

[7] Test‐taking strategies for nursing students. Denver College of Nursing website. Published February 23, 2024. Accessed May 4, 2025. https://www.denvercollegeofnursing.edu/blog/tips‐advice/nursing‐school‐test‐taking‐strategies.html

[8] Test‐taking strategies for nursing school: SHORT. Level Up RN YouTube page. October 21, 2023. Accessed May 4, 2025. https://www.youtube.com/watch?v=ndQXRgv8NRk

[9] Vera M . 75 NCLEX test taking tips and strategies. Nurseslabs. Updated May 27, 2025. Accessed June 1, 2025. https://nurseslabs.com/nclex‐tips/

[10] Olyan‐Yaw M . How to pass nursing school tests/exams. Elsevier Student Hub. Accessed May 4, 2025. https://evolve.elsevier.com/studentlife/blog‐post/a‐guide‐to‐taking‐nursing‐school‐tests‐exams/

[11] Test‐taking tips and strategies for college or nursing school. RegisteredNurseRN YouTube page. March 21, 2016. Accessed May 4, 2025. https://www.youtube.com/watch?v=cwh9Q_LgtiQ

[12] NP learning – test taking strategies: conquering your NP board exam. Sarah Michelle NP Reviews YouTube page. January 22, 2023. Accessed May 4, 2025. https://www.youtube.com/watch?v=VnBFHf4oBKQ

[13] Wiesen K . 10 test‐taking strategies for nurses. Minority Nurse website. Published December 25, 2024. Accessed May 4, 2025. https://minoritynurse.com/10‐test‐taking‐strategies‐for‐nurses

[14] Ahn WK . Thinking 101: How to Reason Better to Live Better. Flatiron Books; 2022.

[15] Merry JW , Elenchin MK , Surma RN . Should students change their answers on multiple choice questions? Adv Physiol Educ. 2021;45(1):182‐190. doi:10.1152/advan.00090.2020 33661053

[16] Tertichny A , Moore CM . The development of the RA‐VING Self‐Trust Instrument (RSTI) across three national samples of adults in the United States. Meas Eval Couns Dev. 2024;57(3):231‐262. doi:10.1080/07481756.2023.2288056

[17] SAMHSA's Trauma and Justice Strategic Initiative . SAMHSA's Concept of Trauma and Guidance for a Trauma‐Informed Approach . Substance and Mental Health Services Administration. US Department of Health and Human Services; 2014. Accessed Feburary 10, 2025. https://library.samhsa.gov/sites/default/files/sma14‐4884.pdf

[18] Thiele DK , Najjar RH . Using trauma informed approaches to enhance teaching and learning. Nurse Educ. Published online April 4, 2025. doi:10.1097/NNE.0000000000001865 40184487

[19] Gunderson RL , Mrozla‐Toscano CF , Mao DM . An instructor's guide for implementing trauma‐informed pedagogy in higher education. J Fac Dev. 2023;37(2):80‐86.

[20] Verschelden C . Bandwidth Recovery: Helping Students Reclaim Cognitive Resources Lost to Poverty, Racism, and Social Marginalization. 1st ed. Routledge; 2017.

[21] Bremner JD . Traumatic stress: effects on the brain. Dialogues Clin Neurosci. 2006;8(4):445‐461. doi:10.31887/DCNS.2006.8.4/jbremner 17290802 PMC3181836

[22] Goleman D . Emotional Intelligence: Why It Can Matter More Than IQ. Bantam Books; 1995.

[23] Giglberger M , Peter HL , Kraus E , et al. Daily life stress and the cortisol awakening response over a 13‐months stress period – findings from the LawSTRESS project. Psychoneuroendocrinology. 2022;141:105771. doi:10.1016/j.psyneuen.2022.105771 35489313

[24] Giotakos O . Neurobiology of emotional trauma. Psychiatriki. 2020;31(2):162‐171. doi:10.22365/jpsych.2020.312.162 32840220

[25] Zhu X , Suarez‐Jimenez B , Lazarov A , et al. Sequential fear generalization and network connectivity in trauma exposed humans with and without psychopathology. Commun Biol. 2022;5(1):1275. doi:10.1038/s42003-022-04228-5 36414703 PMC9681725

[26] Suarez‐Jimenez B , Albajes‐Eizagirre A , Lazarov A , et al. Neural signatures of conditioning, extinction learning, and extinction recall in posttraumatic stress disorder: a meta‐analysis of functional magnetic resonance imaging studies. Psychol Med. 2020;50(9):1442‐1451. doi:10.1017/S0033291719001387 31258096 PMC9624122

[27] Eilers H , Aan het Rot M , Jeronimus BF . Childhood trauma and adult somatic symptoms. Psychosom Med. 2023;85(5):408‐416. doi:10.1097/PSY.0000000000001208 37097117 PMC10241439

[28] McCluney CL , Robotham K , Lee S , Smith R , Durkee M . The costs of code‐switching. Harv Bus Rev. Published online November 15, 2019. Accessed September 14, 2023. https://hbr.org/2019/11/the‐costs‐of‐codeswitching

[29] Dickens DD , Powell CM , Tambedou T , Woodruff K , Bailey L . The influence of gendered racial identity centrality on gendered racism and identity shifting among Black undergraduate women at a HBCU. J Black Psychol. 2023;49(6):856‐867. doi:10.1177/00957984221150049

[30] Mullainathan S , Shafir E . Decision making and policy in contexts of poverty. In: Shafir E , ed. The Behavioral Foundations of Public Policy. Princeton University Press; 2013. doi:10.1515/9781400845347-020

[31] Patton JD , Caffrey T . Hispanic students in higher education: a case for trauma informed approaches. J Hum Behav Soc Environ. 2023;33(5):711‐723. doi:10.1080/10911359.2022.2089311

[32] A*ddressing Racism and Advancing Equity in Midwifery Education: A Program Content Toolkit for Action* . American College of Nurse Midwives; 2024. Accessed August 13, 2025. https://midwife.org/wp‐content/uploads/2024/09/addressing_racism_and_advancing_equity_in_midwifery_education‐a_program_content_toolkit_for_action.pdf

[33] McIntosh P . White privilege: unpacking the invisible knapsack. The SEED Project website. Published July/August 1989. Accessed May 4, 2025. https://www.nationalseedproject.org/key‐seed‐texts/white‐privilege‐unpacking‐the‐invisible‐knapsack

[34] Sue DW , Calle CZ , Mendez N , Alsaidi S , Glaeser E . Microintervention Strategies: What You Can Co to Disarm and Dismantle Individual and Systemic Racism and Bias. Wiley; 2021.

[35] Tatum BD . “*Why Are All the Black Kids Sitting Together in the Cafeteria?”: And Other Conversations about Race* . Basic Books; 2017.

[36] Crabtree M , Thompson KL , Robertson EM . Answer changing behaviors and performance in a first‐year medical gross and developmental anatomy course. HAPS Educ. 2024;28(2):19‐27.

[37] Schraw G , Dennison RS . Assessing metacognitive awareness. Contemp Educ Psychol. 1994;19(4):460‐475. doi:10.1006/ceps.1994.1033

[38] Fadillah SM , Ha M , Nuraeni E , Indriyanti NY . Exploring confidence accuracy and item difficulty in changing multiple‐choice answers of scientific reasoning test. Malays J Learn Instr. 2023;20(2):319‐341. doi:10.32890/mjli2023.20.2.5

[39] Kruger J , Wirtz D , Miller DT . Counterfactual thinking and the first instinct fallacy. J Pers Soc Psychol. 2005;88(5):725‐735. doi:10.1037/0022-3514.88.5.725 15898871

[40] Miller J , Ruppel K , Kane C , Schloemer B , Power P , Owens H . Analyzing traditional and accelerated nursing student exam answer changing behaviors: a retrospective study. Nurse Educ Today. 2023;120:105602. doi:10.1016/j.nedt.2022.105602 36334544

[41] Beebe SL , McNelis AM , El‐Banna M , Dreifuerst KT . Reflecting on diagnosis: the Metacognitive Diagnostic Reasoning Model©. J Am Assoc Nurse Pract. 2024;36(12):711‐718. doi:10.1097/JXX.0000000000001018 39631043

[42] Hrelic DA . Test‐taking tips. American Nurse website. Published March 6, 2018. Accessed May 4, 2025. https://www.myamericannurse.com/test‐taking‐tips

[43] American Midwifery Certification Board 2022 Annual Report . American Midwifery Certification Board; 2023. Accessed May 10, 2025. https://www.amcbmidwife.org/docs/default‐source/annual‐reports/2022‐amcb‐annual‐report.pdf

[44] Exam results ‐ 2020 to 2024. National Certification Corporation website. Published 2025. Accessed May 4, 2025. https://www.nccwebsite.org/certification‐exams/other‐helpful‐information/statistics

[45] AANPCB 2024 Certification Statistics . American Academy of Nurse Practitioners Certification Board; 2025. Accessed May 4, 2025. https://www.aanpcert.org/resource/documents/annual/AANPCB%202024%20Certification%20Statistics.pdf

[46] AANPCB 2023 Annual Report . American Academy of Nurse Practitioners Certification Board; 2024. Accessed May 4, 2025. https://www.aanpcert.org/resource/documents/annual/2023%20Annual%20Report.pdf

[47] AANPCB 2022 Statistics . American Academy of Nurse Practitioners Certification Board; 2023. Accessed August 13, 2025. https://www.aanpcert.org/resource/documents/AANPCB%202022%20Statistics.pdf

[48] 2024 ANCC Certification Data . American Nurses Credentialing Center; 2025. Accessed May 4, 2025. https://www.nursingworld.org/globalassets/docs/ancc/ancc‐cert‐data‐website.pdf

[49] 2023 ANCC Certification Data . American Nurses Credentialing Center; 2024. Accessed May 4, 2025. https://nursing.buffalo.edu/content/dam/nursing/PDFs/ancc‐cert‐data‐2023‐website.pdf

[50] Kaplan . AANPCB vs. ANCC FNP Certificaiton Exams. Accessed May 4, 2025. https://www.kaptest.com/study/nursing/aanpcb‐fnp‐exam‐vs‐ancc‐fnp‐exam/

